# Coronary artery aneurysm combined with other multiple aneurysms at multiple locations: A case report and systematic review

**DOI:** 10.1097/MD.0000000000009230

**Published:** 2017-12-15

**Authors:** Li-Cheng Jiang, Jia-Yu Cao, Mao Chen

**Affiliations:** Department of Cardiology, West China Hospital, Sichuan University, Chengdu, Sichuan, China.

**Keywords:** case report, coronary aneurysm, multiple aneurysms, systematic review, systemic aneurysms

## Abstract

**Background:**

Coronary artery aneurysm (CAA) with concomitant aneurysms at multiple sites is quite unusual and rare. The characteristics and the etiology of this phenomenon are unknown.

**Methods:**

Herein, we present a case with right coronary aneurysm with concomitant abdominal aorta as well as right renal artery aneurysm. A systematic review of the literatures regarding CAA with other coexisting aneurysms at multiple locations was also conducted on Medline and Embase databases.

**Results:**

A total of 76 patients (male gender: 58; age: 37.4 ± 26.5) including the present case were included in the final study. The most common etiology of CAA with multiple aneurysms was Kawasaki (43.3%) and atherosclerotic disease (16.4%). CAA was the most frequently found at the right coronary artery (62.7%), following, left anterior descending (51%), left main (43.1%), and left circumflex (35.3%). The most common concomitant aneurysms were abdominal aorta (52.6%) and iliac artery (50%). In addition, 60.5% of the patients had an involved bilateral peripheral artery.

**Conclusion:**

CAA with coexisting systemic aneurysms in multiple sites is quite rare. And it usually involves multiple aneurysms at the coronary and bilateral peripheral arteries simultaneously. Currently, there are no general consensus regarding the clinical characteristics, diagnostic method, and treatment of these cases.

## 1 Introduction

Coronary artery aneurysm (CAA) is currently defined as a coronary artery dilatation > 1.5 times the diameter of the normal adjacent segments or the diameter of the patient's largest coronary vessel,^[1]^ and was reported to be 1 to 4% of coronary angiography findings.^[2]^ The reports of CAA combined with an additional aneurysm are quite common. However, CAA with concomitant aneurysms at multiple locations are quite unusual and rare,^[3,4]^ and is mostly limited to case reports and small case series. Thus, a systematic review of literatures regarding CAA with coexisting aneurysms at multiple locations was performed. In addition, a case of a patient with right CAA, abdominal aorta, and right renal artery aneurysm from our center was also included.

## 2 Case

A 62-year-old female patient with a history of mild hypertension for over 10 years presented with paroxysmal exertional chest pain for 1-week, which progressively worsened over the past 12 hours, was admitted to our center. There was no significant positive physical findings. The electrocardiogram (ECG) showed slight ST segment elevation of leads III and avF during chest pains. Her echocardiography revealed a normal functioning heart (left ventricular ejection fraction: 65%) with no abnormal wall motion or cardiac enlargement. Her troponin level was 967.6 ng/L (normal, ≤14 ng/L).

**Figure 1:**
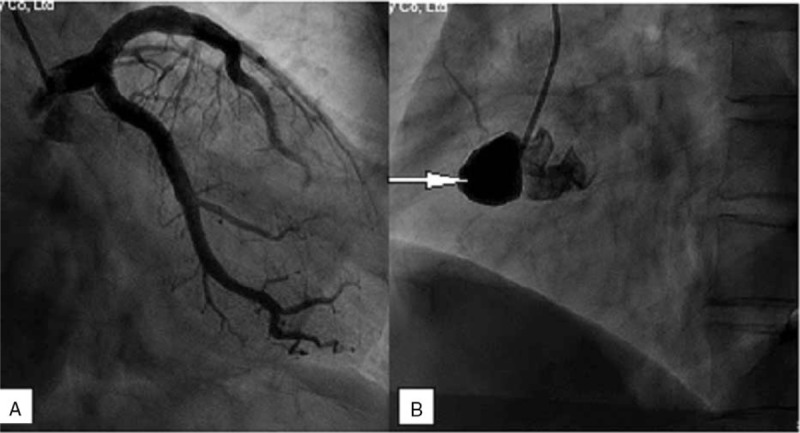
(A) No obvious abnormality was found in the left coronary angiography. (B) A coronary artery aneurysms in the ostium of the right coronary artery (RCA) and the RCA cannot be seen except for the conus branch. RCA = right coronary artery.

**Figure 2:**
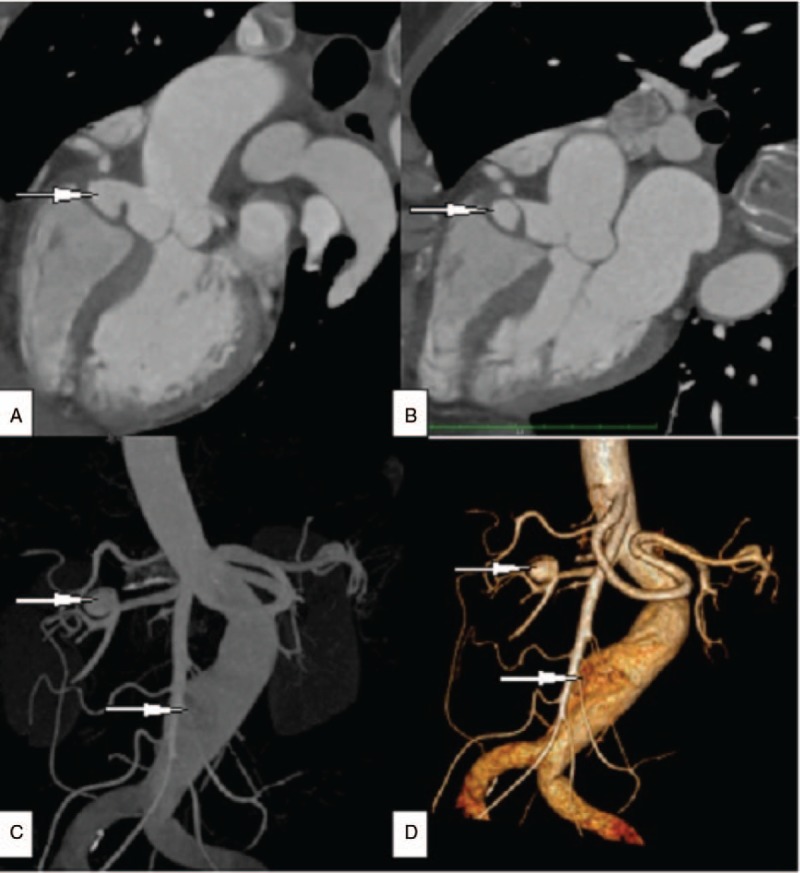
(A, B) CTA showed an aneurysm in the opening of RCA and leads to a severe stenosis of the ostium of the right coronary. (C, D) An aneurysm in the distal segment of right renal artery and an aneurysmal dilatation in the third lumbar plane of abdominal aorta in the CTA. CTA = computer tomography angiography RCA = right coronary artery.

The patient underwent coronary angiography (CAG) due to the possibility of an acute non-ST segment elevation myocardial infarction. It revealed an aneurysm at the ostium of the right coronary artery (RCA), which resulted in total occlusion of the proximal segment of the RCA, except for the conus branch (Fig. 1). There was no obvious obstruction or pathological lesions in the left coronary artery. Next, computer tomography angiography (CTA) of the head, neck, thoracic, and abdominal aorta was performed to rule out other potential arterial diseases. It revealed an aneurysm in the distal segment of right renal artery as well as an aneurysmal dilatation in the third lumbar plane of abdominal aorta, and no obvious abnormalities in the head and neck arteries (Fig. 2). The patient's C reactive protein, serum creatinine, immunoglobulin, antinuclear antibody, anticardiolipin antibody, and antineutrophil cytoplasmic autoantibody levels were all normal.

Due to the patient's ongoing angina and the presence of an aneurysm at the ostium of RCA, which resulted the total occlusion of the proximal segment of the RCA, the patient eventually underwent aneurysmal ligation with a concomitant distal bypass graft. However, the renal artery and abdominal aortic aneurysms were conservatively treated due to no related symptoms. The patients made an uneventful recovery and were discharged on the tenth day. At 11-month follow-up, the patient was alive and well without limitations in her daily life.

## 3 Materials and methods

A systematic review was performed on PubMed and Embase to identify literatures describing CAA with other coexisting aneurysms at multiple locations (more than 2 sites) with the following keywords: (“coronary aneurysm” OR “coronary artery aneurysm,” OR “aneurysmal coronary artery”) AND (“brain” OR “intracranial” OR “anterior communicating artery” OR “cerebral” OR “vertebral” OR “carotid” OR “subclavian” OR “axillary” OR “brachial” OR “radial” OR “ulnar” OR “aorta sinus” OR “aorta” OR “pulmonary” OR “celiac axis” OR “mesenteric” OR “splenic” OR “hepatic” OR “renal” OR “iliac” OR “femoral” OR “popliteal” OR “multiple” OR “systemic” OR “diffuse”).

All published case reports and case series describing CAA with coexisting aneurysms ≥2 were included. There were no language or time restrictions. Cross-referencing was also performed with the included studies to identify potentially relevant studies. Data were managed with Microsoft Excel. Data are expressed as mean ± SD and percentages.

A total of 1448 studies were initially identified. After excluding unrelated studies and duplicates, 60 articles were included. After cross-referencing from the included studies, a total of 61 articles describing CAA with multiple coexisting aneurysms were finally included,^[3–63]^ with a total of 76 patients (age: 37.4 ± 26.5 years; male: 58 [76.3%]). The patients’ ages ranged from 7 weeks to 81 years, and 24 patients were less than or equal to 18 years of age.

## 4 Results

Out of the 76 patients, 67 of the cases had described the etiology of the aneurysm, which were Kawasaki disease (n = 29; [43.3%]), atherosclerosis (n = 11 [16.4%]), IgG4-related disease (n = 3), Marfan's syndrome (n = 2), Behcet's disease (n = 2), systemic lupus erythematosus (SLE) (n = 2), hypereosinophilic syndrome (n = 2), and autosomal dominant polycystic disease (ADPKD) (n = 2). Other etiologies include fibromuscular dysplasia (FMD), hyperhomocysteinemia, giant cell arteritis, unclear inflammatory disease, autosomal dominant inheritance of familial aortic aneurysm and/or dissection, Ehlers–Danlos syndrome type IV, myxoma-related disease, chronic active Epstein–Barr virus infections (CAEBV), mycobacterium bovis, takayasu arteritis, angiolymphoid hyperplasia with eosinophilia (ALHE) associated with mycosis fungoid, and polyarteritis nodosa only occurred in 1 patient (see Table 1).

**Table 1:**
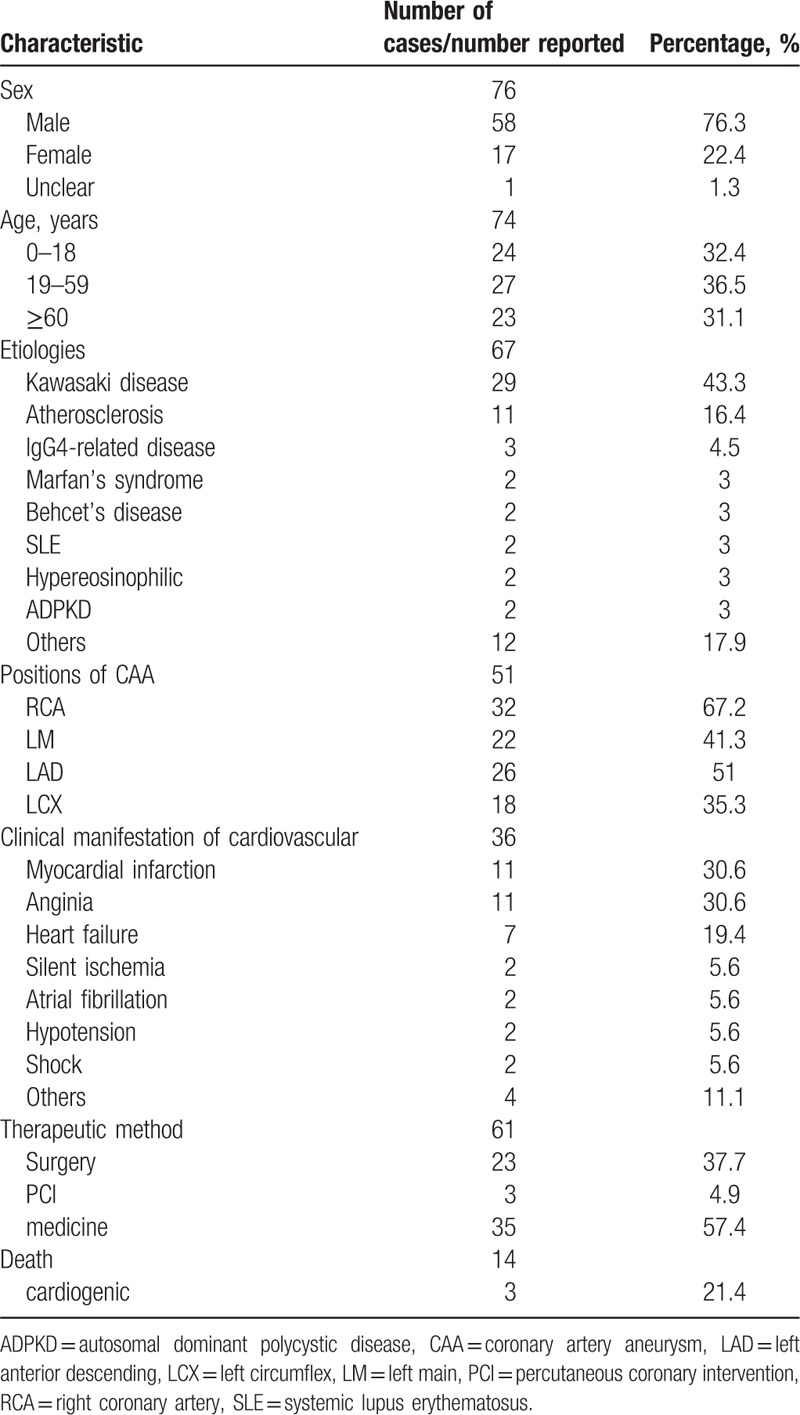
Patient characteristics and analysis of selected characteristics of CAA.

Out of the 76 patients, 53 (69.7%) of them had aneurysms in both coronary arteries, and 51 patients identified the concrete site of the CAA. Out of the these, 32 occurred on the RCA (62.7%) and 22 occurred on the left main (LM) coronary artery (43.1%), 26 occurred on the left anterior descending (LAD) coronary artery (51%), 18 occurred on the left circumflex (LCX) coronary artery (35.3%), and 35 (68.6%) had multiple coronary artery aneurysms. Also 47 patients had description of the morphology of aneurysms through detail description or imaging studies. Around 21 of the patients were presented with saccular aneurysm and 18 patients were presented with fusiform anerysm, and 8 patients had both type. As well as that, only 48 individual cases had differentiated whether if the luminal stenosis was in the proximal or distal segment of the aneurysm. A total of 36 patients had concomitant cardiovascular comorbidities which includes myocardial infarction (n = 11), angina pectoris (n = 11), heart failure (n = 7), atrial fibrillation (n = 2), silent myocardial ischemia (n = 2), hypotension (n = 2), shock (n = 2), complete heart block (n = 1), thromboembolism (n = 1), syncope (n = 1), and murmur (n = 1) (see Table 1). Out of the 76 patients with coronary aneurysms, 56 of the cases were diagnosed with CAG. The remaining patients were confirmed by means of echocardiography, computed tomographic angiography, and cardiac magnetic resonance (CMR), or autopsy.

**Figure 3:**
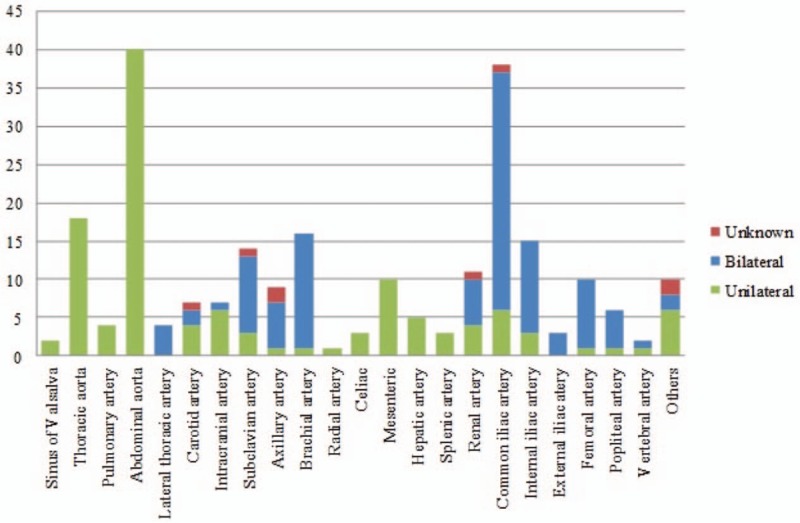
The locations of the aneurysms outside of coronary.

Concomitant vascular aneurysm sites were mostly abdominal aorta (n = 40, 52.6%), common iliac artery (n = 38, 50%), and thoracic aorta (n = 18, 23.7%). The remaining concomitant vascular aneurysm sites included were: brachial artery (n = 16); internal iliac artery (n = 15); subclavian artery (n = 14); renal artery (n = 11); axillary artery (n = 10); mesenteric artery (n = 10); femoral artery (n = 10); carotid artery (n = 7); intracranial artery (n = 7); popliteal artery (n = 6); pulmonary artery (n = 4); lateral thoracic artery (n = 4); external iliac artery (n = 3); celiac artery (n = 3); and finally vertebral artery (n = 2). Other sites of aneurysm include occipital artery, aorto-pulmonary collateral artery, intrapulmonary collateral artery, superficial popliteal artery, vertebral, internal mammary artery, profunda brachii artery, bronchial artery, thyrocervical trunks, posterior tibial artery, left ulnar artery, radial artery, and brachiocephalic artery. Simultaneous bilateral peripheral artery aneurysm was present in 46 of 76 patients (60.5%) (Fig. 3).

Among the 76 reported patients with coronary aneurysms, 61 patients received treatments (Table 1). Surgical treatment including coronary artery bypass grafting (CABG) and aneurismal resection or ligation was carried out in 23 (37.7%) individuals. Percutaneous coronary intervention (PCI) was received in 3 (4.9%) patients, and conservative medical therapy (aspirin or warfarin) or etiological treatment of primary diseases were given to 35 (57.4%) patients.

All-cause mortality occurred in 14 (18.4%) patients. Three individuals died of cardiogenic factors, other causes of mortality include multiple organ failure (n = 2), sepsis (n = 2), progressive respiratory failure (n = 2), and ruptured aneurysm inducing uncontrollable hemorrhage occurred in 3 patients.

## 5 Discussion

Since Bougon first described CAAs in 1812,^[64]^ many literatures regarding aneurysmal coronary disease have since been published. However, CAA with concomitant vascular aneurysms at multiple locations is rare. The clinical presentations and pattern of risk factors seen in individual cases in the present study would suggest that this disease remains unclear.

In the current study, the ratio of males with CAAs and concomitant aneurysms at multiple vascular sites was 76.3%, which was lower compared to the Coronary Artery Surgery Study (CASS).^[1]^ Atherosclerosis was the most common cause of isolated-CAA, which occupied over half of the total reported cases. Though, atherosclerotic disease usually occurs in older patients,^[65]^ CAAs could still take place at most ages, even in younger patients. Thus, suggesting the causes of CAA are most likely multiple. Other common causes of CAA include Kawasaki disease, mycotic, and infectious septic emboli, Marfan's syndrome, connective tissue diseases, or arteritis.^[66]^

The current study found Kawasaki disease as the most common etiology of CAA with concomitant aneurysms at multiple vascular sites, which occupied 43.3% of the total reported cases in our review. Furthermore, the majority of patients were mostly children and adolescents in this group. The second was atherosclerosis, which occupied 16% of the patients. Other remaining causes include SLE, Behcet's disease, and interestingly, most of the patients were females in these patients.

In isolated-CAA, site of aneurysmal disease was more frequently at the RCA (40%), than following, LAD (29%), LCX (24%), and LM (7%).^[67]^ In the current study, the frequency of aneurysm at the LM was 43.1% and 68.6% of the total study had multiple coronary artery aneurysms. Of which, 62.7% at the RCA, 51% at the LAD, and 35.3% at the LCX. The reason for this novel finding is unclear, and merits a further attention.

Depending on the size and, number of aneurysms, or stenosis at the proximal or distal coronary segment of aneurysm, the clinic features of each patients varied. In patients with isolated-CAAs, common clinical manifestations include coronary heart diseases, myocardial infarction, heart failure, thrombotic disease, and syncope.^[68]^ Similarly, the current review demonstrated that the 3 most common clinical features of CAA patients with multiple vascular aneurysms were myocardial infarction, angina pectoris, and heart failure. The main method for diagnosis of CAA was CAG in the current study. However, the use of CTA or CMR is likely to play a key role in the diagnosis of CAAs because they have rapid, noninvasive, and reliable characteristics.

The most common concomitant vascular aneurysm with CAA was abdominal aorta (52.6%). This is likely due to the fact that CAA and abdominal aortic aneurysm (AAA) have similar histological and clinical features.^[69]^ The second was the common iliac artery, which occurred in half of the total reported cases, and third was thoracic aorta (23.7%). Other frequent sites include in brachial, subclavian, internal iliac, renal, axillary, and mesenteric arteries. It was interesting to note that pulmonary and distal peripheral arteries were rarely involved. In the current study, 60.5% of the patients had multiple peripheral arterial aneurysms in a mirror-image pattern across the sagittal plane. Heran and Hockley et al^[33]^ hypothesize that this symmetry in aneurysm distribution was associated with systemic vasculitis, which resulted in the pathological changes in the adjacent peripheral arteries or blood pressure.

Currently, there is no general consensus regarding the treatment of CAAs or CAA with multiple systemic aneurysms. Treatment strategies depend on the presence and degree of coronary artery stenosis, etiology of CAAs, age, as well as accompanying comorbidities. The main treatment of choice includes surgical CABG and aneurismal resection or ligation, PCI, or conservative drug treatment (anticoagulant and etiological treatment).^[70,71]^

According to the CASS registry, CAA patients with prior myocardial infarction or coronary heart disease were associated with increased risk of mortality.^[1]^ In our review, unfavorable prognosis of patients with CAA and multiple vascular aneurysms also depends largely on the location, size, and extent of stenosis of the concomitant aneurysm, as well as the risk of thrombosis. Interestingly, only 3 cases in the 14 dead patients died of rupture of the aneurysms, most died of comorbidities induced to cardiac death, respiratory, or multiple organ failure. Therefore, we should pay attention to the comorbidities and the systemic damage of the diseases in addition to the aneurysms themselves.

## 6 Conclusion

In conclusion, cases CAA with multiple systemic aneurysms are quite rare. The clinical characteristics of these patients were somewhat similar to isolated-CAAs. However, multiple vascular aneurysms were frequently involved in bilateral arteries in these patients. Currently, there are no general consensus regarding the clinical features, diagnostic method, and treatment of these cases. The anatomic morphology of the aneurysm and clinical characteristics should guide the specific treatment strategy of each individual cases.

## 7 Limitations

This review failed to describe the morbidity and mortality in the crowd due to limited data.

## 8 Consent

Written informed consent was obtained from the patient for publication of this case report and accompanying images. The systematic review needs no ethical approval.

## Acknowledgments

We thank the combination and support of the patient in this study, and Dr Yi-jian Li and Dr Fang-yang Huang for assisting in preparation of this manuscript
